# How unemployment scarring affects skilled young workers: evidence from a factorial survey of Swiss recruiters

**DOI:** 10.1186/s12651-018-0239-7

**Published:** 2018-06-29

**Authors:** Lulu P. Shi, Christian Imdorf, Robin Samuel, Stefan Sacchi

**Affiliations:** 1 Social Research and Methodology Group, University of Basel, Petersgraben 27, 4051 Basel, Switzerland; 2 Department of Sociology and Political Science, Norwegian University of Science and Technology, Dragvoll, bygg 10, 7491 Trondheim, Norway; 3 Université du Luxembourg, Maison des Sciences Humaines, 11, Porte des Sciences, 4366 Esch-sur-Alzette, Luxembourg; 4 Institute of Sociology, University of Bern, Fabrikstrasse 8, 3012 Bern, Switzerland

**Keywords:** Recruitment, Youth unemployment, Scarring, Hiring, Factorial survey, Switzerland, J64/E24 Unemployment, M51 Firm Employment Decisions, C93 Field Experiments

## Abstract

We ask how employers contribute to unemployment scarring in the recruitment process in the German-speaking part of Switzerland. By drawing on recruitment theories, we aim to better understand how recruiters assess different patterns of unemployment in a job candidate’s CV and how this affects the chances of young applicants being considered for a vacancy. We argue that in contexts with tight school-work linkage and highly standardised Vocational Education and Training systems, the detrimental effect of early unemployment depends on how well the applicant’s profile matches the requirements of the advertised position. To test this assumption, we surveyed Swiss recruiters who were seeking to fill positions during the time of data collection. We employed a factorial survey experiment that tested how the (un)employment trajectories in hypothetical young job applicants’ CV affected their chances of being considered for a real vacancy. Our results show that unemployment decreases the perceived suitability of an applicant for a specific job, which implies there is a scarring effect of unemployment that increases with the duration of being unemployed. But we also found that these effects are moderated by how well the applicant’s profile matches the job’s requirements. Overall, the worse the match between applicant’s profile and the job profile, the smaller are the scarring effects of unemployment. In sum, our findings contribute to the literature by revealing considerable heterogeneity in the scarring effects of unemployment. Our findings further suggest that the scarring effects of unemployment need to be studied with regard to country-specific institutional settings, the applicants’ previous education and employment experiences, and the job characteristics.

## Introduction

In recent years, a broad body of literature has shed light on how unemployment affects (re-)integration in the labour market and has investigated under what conditions the so-called scarring effects of unemployment may occur. The effects of unemployment on later employment chances vary from country to country and occupation to occupation (Grip and Wolbers 2006). They depend on factors such as cyclical changes in the overall economic condition and institutional school-work linkage (Breen 2005; Gangl 2002; Müller and Gangl 2003; Wolbers 2007). With regard to youth unemployment, it is important to ask in what contexts unemployment matters for individuals’ later labour-market outcomes. Demand-side studies have focused on recruiters’ perceptions of unemployment and how this can influence their hiring decisions. Applicants’ features, such as the duration of unemployment (Kroft et al. 2013; Oberholzer-Gee 2008), and characteristics of the vacancy, such as the skill level of the job (Bonoli 2014), have both been shown to have determinant influences on hiring outcomes.

Country-specific classifications of education systems and labour markets are often too crude to capture recruiting dynamics that vary greatly across different types and levels of jobs within countries. Depending on their type and level, different occupations are connected to different educational tracks. The tightness of the school-work linkage varies accordingly, which in turn leads to varying recruitment strategies. In this article, we focus on the regional labour-market context of German-speaking Switzerland, which is characterised, like Germany, by a high degree of vocational specificity and standardisation in the school-to-work transition system (Imdorf et al. 2017; Shavit and Müller 1998; Müller and Gangl 2003; Stalder and Nägele 2011). Compared to other OECD countries, the youth unemployment rate is relatively low in Switzerland, and it was also at a comparably low level after the recent Great Recession. In the fourth quarter of 2015, 9.8% of young people between 15 and 24 were unemployed (OECD 2015; according to the ILO definition of unemployment). The proportion of skilled young workers at the entry level who have been unemployed long-term (0.1% in September 2012) is lower than that of skilled older workers (0.4% in September 2012) (Sacchi 2012: 9). This picture has remained stable since 2004, showing a low share of young workers remaining unemployed over 1 year (ibid.). We are thus analysing the contextual significance of unemployment scarring in a country with a stable economy and relatively low unemployment figures compared to many European countries.

To understand the consequences of early unemployment, we draw on signalling theory (McCormick 1990; Spence 1973) and human capital theory (Becker 1964; Pissarides 1992). We argue that in contexts with a structural school-to-work transition such as in Switzerland, the detrimental effect of early unemployment depends on how well the applicant’s profile matches the requirements of the advertised position. By conducting a factorial survey experiment, we have aimed to overcome the challenge, addressed in recent recruitment studies, of simultaneously taking recruiters’ and applicants’ characteristics into account. Our methodological design enables us to account for applicants’ characteristics and the recruiters’ side by controlling for the match between the applicants’ profiles and the job requirements. The factorial experiment is built upon hypothetical applications tailored to real-world job vacancies, which are evaluated by the recruiters in charge of filling the selected vacant positions. This unique experimental design helps maximise external and internal validity.

In the following, we first discuss the findings of previous recruitment studies on the scarring effects of unemployment (Sect. 2). In Sect. 3, we present the theoretical framework of our study, from which we derive our hypotheses. Section 4 describes the data and methods applied. The results are presented in Sect. 5, followed by their discussion in Sect. 6. A conclusion is given at the end.

## Unemployment scarring in previous recruitment studies

Studies on recruitment have been attempting to examine the impact of unemployment on future labour-market outcomes. Different studies have investigated the link between the duration of unemployment and later employment outcomes. This association is referred to as duration dependence in the labour-market literature (Eriksson and Rooth 2014; Luijkx and Wolbers 2009; Oberholzer-Gee 2008; Kroft et al. 2013; van den Berg and van Ours 1996, 1999). While the majority of studies have found evidence for a negative duration dependence (Abbring et al. 2001; van den Berg and van Ours 1996, 1999; Kroft et al. 2013; Luijkx and Wolbers 2009), the results of other studies do not support an association between the duration of unemployment and future employment outcomes (Farber et al. 2015; Nunley et al. 2017). Yet other studies have found duration dependence for some groups but not for others, suggesting a high degree of heterogeneity in how unemployment relates to future career trajectories (Eriksson and Rooth 2014).

Oberholzer-Gee (2008) tested the impact of different lengths of unemployment, from 6 months up to 30 months, on future employment chances by sending fictitious CV to recruiters in Switzerland and measuring the callback rate. The author found evidence that employers recruiting administrative assistants in Switzerland prefer the short-term unemployed (6 months) to currently employed applicants. Kroft et al. (2013) found the same effect in a correspondence-testing study that was carried out in a hundred cities in the United States with different labour-market tightness. The results of both studies suggest that employers prefer the currently unemployed because they can start working immediately. Nevertheless, the callback rate eventually decreases when the duration of unemployment exceeds a certain length (8 months in the US study and 2 years in the Swiss study). Van den Berg and van Ours (1996, 1999) investigated how the impact of unemployment on later career chances varies between groups of people with different socio-economic backgrounds by using national data provided by the US Bureau of Labor Statistics and French public employment offices. The authors found evidence that the duration dependence varies for different ethnic groups (1999) and between women and men (1996).

In contrast to the above studies, Farber et al. (2015) could not find support for duration dependence in their audit study carried out across eight US cities in 2012 and 2014 with fictitious CV showing different durations of unemployment. Collecting data in different geographical areas and at different times made it possible to account for varying unemployment rates. But no significant correlation between the duration of unemployment and callback rate could be found across cities with differing labour markets. Similarly, in a recent correspondence study carried out by Nunley et al. (2017) in seven large US cities, the authors did not find any significant differences between the callback rates for fictitious CV showing 3, 6, or 12 months of unemployment, independent of geographical location. And a Swedish study carried out by Eriksson and Rooth (2014) showed that duration dependence can vary between different job segments. Whereas a current period of unemployment of at least 9 months has a negative impact on callback rates in medium- and low-skill jobs, they did not find any negative duration dependence for high-skill jobs. The differences in the findings and the difficulty in determining whether and how the duration of unemployment impacts future labour-market outcomes suggests that context matters and moderates the consequences of unemployment.

Various studies have identified many factors that affect the employment chances of applicants who have experienced unemployment. They range from educational background and work experience to the duration of unemployment and the job requirements. But previous studies have neglected how the effect of unemployment varies as a function of the match between the applicant’s characteristics and the job requirements depending on the institutional setting. Although there is a large body of recruitment studies both on (mis-)match and on unemployment, to our knowledge there are only two recruiter-side studies that compare both effects (Baert and Verhaest 2014; Nunley et al. 2017). Both studies apply correspondence testing to investigate the consequences of previous job mismatch and unemployment on subsequent employment careers. They reconstruct previous job mismatch with fictitious CV that present work experience that is at a lower level than the applicant’s earlier qualifications obtained through education. Such an approach makes it possible to test recruiters’ evaluations of CV with a poor correspondence between the type of education and previous job experience. But it does not provide insights into the consequences of a *mismatch* between the requirements of a vacant position and the educational background and job experience of the applicants. Furthermore, this approach does not help elucidate how the effects of unemployment are moderated by a mismatch between the applicant’s profile and the job requirements. We will thus contribute to the debate on mismatch and unemployment in two ways: first, by showing how unemployment scarring is moderated by a (mis-)match between the applicant’s profile and the job requirements. Second, by differentiating between mismatch in occupation specificity and mismatch in educational level, which we address as horizontal and vertical mismatch, respectively.

## Theoretical framework and hypotheses

We draw on human capital theory and signalling theory to derive our hypotheses. Both theoretical approaches have been extensively used to study the scarring effects of unemployment on hiring chances (Arulampalam et al. 2000; Lockwood 1991; Omori 1997). From the perspective of *human capital theory*, skill and knowledge are accumulated during time spent in education or at work (Becker 1964; Pissarides 1992). The time invested in education and work experience can therefore be seen as an indicator of the productivity of an applicant. This implies that times of inactivity can be seen as missed opportunities to gain human capital. Also, previously acquired skills can be lost if they are not practiced and updated through working (Blanchard and Summers 1986; Phelps 1970; Pissarides 1992). Longer durations of unemployment therefore lead to greater depreciations in expected potential human capital.

*Signalling theory* starts from the premise that hiring is a situation of imperfect information since recruiters cannot observe the productivity of applicants at the time of the hiring decision. Because it would be difficult, time-consuming, and costly to directly assess applicants’ productivity, recruiters draw on visible cues, the so-called signals (Spence 1973), to derive an estimate of productivity. Applicant features such as education credentials, school grades, and previous job titles serve as signals for the applicants’ unknown productivity and trainability (Di Stasio 2014). In addition, we may assume that an applicant’s productivity also depends on the quality of the job-worker match (see Oyer and Schaefer 2011: 1786–87), suggesting that signals will also be used to evaluate how well the applicant’s profile meets the job requirements. Furthermore, information can also be drawn from more implicit cues such as gaps in the CV or frequent job changes, and it has been argued that unemployment can have a negative impact on the chances of finding a job and on future wages (Arulampalam et al. 2000, 2001; Schmieder et al. 2016). Studies investigating the signalling value of unemployment have found that employers might associate unemployment with low motivation, low productivity, and undesirable personality traits (Atkinson et al. 1996; Bonoli 2014), resulting in a higher risk of workplace disruption (Devins and Hogarth 2005). As a consequence, unemployment can lead to lower rates of being able to find a job (Eriksson and Lagerström 2006).

Similar to human capital assumptions, the signalling value of unemployment can differ with the length of unemployment: longer periods of unemployment may have a stronger negative signal value than shorter periods. Some studies suggest that depending on the economic context and occupational field, short current unemployment does not necessarily decrease the chances of being hired (Oberholzer-Gee 2008). The signal of unemployment must thus be understood within specific contexts. The characteristics of the applicants, the features of the advertised jobs, and the country-specific institutional context must be accounted for.

In sum, we argue that the chance of being considered for a job is negatively associated with the presence and duration of unemployment. *All else being equal, recruiters will evaluate applicants with long periods of unemployment less favourably than those with short unemployment or without any unemployment* (*hypothesis H1*). Switzerland seems especially suitable for analysing the signalling value of unemployment since it has a strong^1^ and strongly standardised VET system with high specificity. This fosters a relatively smooth transition into the labour market because employers trust VET credentials, which facilitates the entrance of VET holders into the labour market (Breen 2005). The tight school-work linkage and low youth unemployment rate suggest that the negative signalling value of unemployment should be more pronounced (Biewen and Steffes 2010) and increase with growing duration of unemployment. If unemployment occurs, the corresponding gaps on a CV will draw recruiters’ attention and be evaluated cautiously.

Depending on the specific qualifications offered by different academic and vocational-training programmes, different skills and knowledge—and hence different types of human capital—are acquired during education. The signalling value of credentials therefore varies substantially across different types of education within a country (Allmendinger 1989). In comparison to the more generic signals of general-education certificates, VET education primarily aims to prepare learners for the labour market by providing them above all with occupation-specific skills, and VET certificates provide standardised information about job applicants’ skills. Hence, for jobs requiring specialised skills, the completion of a suitable VET certificate is a crucial signal for the perceived productivity of a job candidate.

However, in addition to occupational credentials, adequate work experience may also matter. Despite the tight education-employment linkage in the occupationally segmented Swiss labour market, VET certificates do not necessarily guarantee a smooth transition from school to work anymore due to increasingly complex work realities and requirements (Salvisberg and Sacchi 2014). As a consequence, employment experiences and further training in the field are often required and gain in importance as sorting criteria in hiring for qualified jobs. Considering the competition between VET-degree holders for skilled jobs, it can be argued that an occupation-specific degree is a necessary but not a sufficient criterion for hiring. We assume that in a country with VET predominant in upper secondary education, both occupation-specific education *and* occupation-specific job experience are the most fundamental requirements in order to be considered for a position. In such a context, we expect recruiters to largely ignore applicants who do not meet these basic requirements. As a consequence, if an application is more or less ineligible due to education and job experience, there will hardly be any leeway left for a negative scarring effect from unemployment. Hence, *the chance of being considered for a job is negatively associated with the presence of periods of unemployment only to the degree that applicants’ profiles meet the fundamental job requirements (hypothesis H2)*.

(Mis-)matches between applicants’ profiles and job requirements can be measured along the horizontal and the vertical axes. A horizontal match is when the applicant has *occupation*-*specific* education and work experience. A vertical match is when the applicant has the required *level* of both education and work experience. As we have argued, the Swiss labour market is strongly occupationally segmented, which means that recruiters attach great importance to applicants’ occupation-specific education and work experience. This leads to the assumption that the horizontal match particularly moderates the scarring effect of unemployment.

Hence, refining the second hypothesis H2, we assume that *the chance of being considered for a job is negatively associated with the presence of periods of unemployment specifically when the applicant has an occupation*-*specific educational background and work experience (hypothesis H3*).

## Data and methods

### Research design

Our data was obtained in the context of the NEGOTIATE project^2^ by conducting a multinational online recruiter survey across five occupational fields—mechanics, finance, nursing, gastronomy, and information technology—and four European countries—Bulgaria, Greece, Norway, and Switzerland (https://negotiate-research.eu/). Within this survey, we embedded a factorial survey experiment and a choice task (Hyggen et al. 2016). The present article draws on the Swiss experimental data of this study based on a factorial survey experiment (FSE). This multidimensional experimental design, which implements vignettes representing hypothetical objects or situations (job applicants’ CV in our case; see Auspurg and Hinz 2015; Jasso 2006; Rossi and Anderson 1982), has been applied in labour-market research investigating behaviours in searching for and accepting jobs (Abraham et al. 2013; Auspurg and Gundert 2015) or evaluating the wage models of organisations (Weibel et al. 2010). In recruitment research, however, FSE has only recently gained attention (Di Stasio 2014; Di Stasio and Gërxhani 2015; Humburg and van der Velden 2015). Conjoint or audit studies are more popular forms for field experiments on recruitment. In such studies, respondents are not aware they are participating in a study, and the effect of the experimental variable(s) are usually measured in terms of the rate of callbacks for job interviews. The major advantage of such a design is its high external validity. But this advantage can backfire if the CV have uncommon combinations of features which may irritate the respondents. Researchers are therefore usually constrained to varying only one or a few of the applicants’ characteristics as the experimental variable(s). In a factorial survey experiment, uncommon trajectories in CV are less problematic. Further, the possibility to vary a large number of applicant features at once is facilitated because each respondent can be asked to evaluate a larger number of CV. This makes it possible to create a pool of hypothetical candidates with a large number of combinations of individual characteristics and to measure their single and joint effects on the recruiters’ evaluations. In addition, it becomes possible to disentangle effects, which tend to covary in a real-world setting (e.g. correlation between certain occupations and gender), by holding the dimensions orthogonal in the experimental design. The experimental variables and their interactions can therefore be tested in isolation from one another, and their effects can be singled out in the analysis. FSE combines the advantages of correspondence testing and conventional surveys: it makes it possible to control for the pool of applicants on the one hand and to access detailed information about the recruiters, the organisational hiring strategies, and recruiting preferences on the other.

### Sampling

To maximise internal and external validity, we choose to sample real-world vacancies using online job ads from German-speaking Switzerland together with the contact information of the recruiters responsible for filling the advertised vacancy. The occupational fields of mechanics, finance (banking and insurance), gastronomy (service personnel), nursing, and information technology (ICT) were chosen for sampling in order to include low-, middle-, and high-skill jobs, gender-mixed and gender-typed jobs, occupations more or less dependent on and linked to technological innovations, and to account for jobs with higher and lower turnover rates.

To ensure that the fictional applicants adequately fit into the profiles of the sampled job vacancies, we developed sampling criteria for each occupational field in order to narrow down the occupation titles based on selected four-digit ISCO-08 codes. The selected job-advertising media were general online job portals, which altogether reached a coverage of at least 95% of the total number of all general online job portals. In addition, vacancies from company websites published on local unemployment agencies’ websites were included in the sampling. The main sampling was carried out on 8th March 2016 by the Swiss Job Market Monitor,^3^ and all the advertised jobs in German-speaking Switzerland that had been posted in the previous 14 days in the preselected job portals were collected. A second sampling was carried out 2 weeks later in which all the job advertisements that had been posted in the previous 7 days were collected. The sampling strategy allowed us to draw a random sample of job vacancies advertised through online job portals for the labour market of German-speaking Switzerland. The vacancies advertised through this channel account for more than half of all vacancies.^4^

Based on this sampling of job ads, 2118 recruiters were contacted, and 739 (35%) of them started the survey.^5^ In comparison to similar recruitment studies relying on a factorial survey experiment (e.g. Liechti et al. 2017: 12% in Switzerland; Damelang and Abraham 2016: 12.5% in Germany), the response rate was relatively high. Response rates within each occupational field varied between 30% (for catering) and 46% (for nursing) for discontinued surveys that provided sufficient information to be included in our analysis (Appendix: Table 5). Further characteristics of the nonrespondents are not accessible because information about the non-responding firms is not available. Table 1 summarises some company-level features of the job sample.

**Table 1:** Frequencies of features of the organisations recruiting (data gathered from the recruiter survey)

Features of the organisations recruiting	Absolute frequencies	Relative frequencies in percentage (%)^a^
*Occupational field*		
Mechanics	129	17.5
Finance	126	17.1
Nursing	205	27.7
Gastronomy (service personnel)	115	15.6
Information technology	164	22.2
*Firm size (number of employees)*		
Less than 50	82	14.0
50 to 249	248	42.3
250 to 999	113	19.3
1000 or more	130	22.2
*Type of organisation recruiting* ^b^		
Recruitment agency	174	25.1
Company (no agency)	501	72.4
Other	17	2.5
*Sector* ^c^		
Public	120	20.4
Private	438	74.5
Other	26	4.4
*Gender of the respondent*		
Female	313	53.8
Male	269	46.2
*Education-level requirement*		
No specific level of education required	31	4.8
At least an upper secondary/VET degree	354	55.2
At least a tertiary degree	256	39.9
*Education-type requirement*		
Occupation-specific education is indispensable	309	47.9
Occupation-specific education is desirable	225	34.9
Occupation-specific education is not necessary	111	17.2

^a^ The percentages of single variables may not add up to 100 per cent due to missing cases. We have included the data from discontinued surveys if the relevant item was answered

^b^ We differentiate between respondents who were working as external recruiters in employment agencies (“recruitment agency”) and respondents who were working for one of the companies that advertised a position to be filled (“company”)

^c^ We differentiate between job positions in the public and the private sector

### Experimental variables

**Fig. 1:**
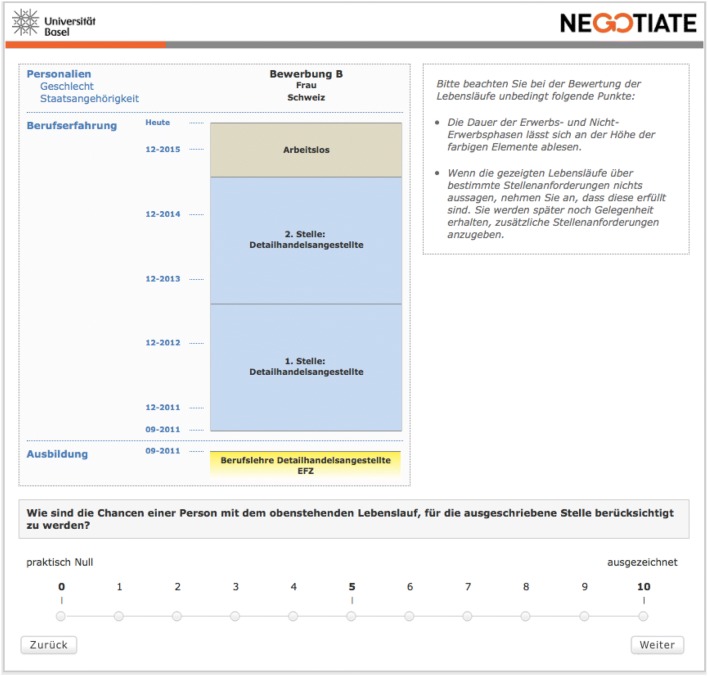
Illustration of a fictive CV

To measure the signalling effect of unemployment and the moderating effects of education and work experience, we set up a 9^1^7^1^2^2^ design with the experimental variables^6^ being *occupation specificity and level of education and work experience* (nine different combinations), *duration and timing of unemployment* (seven different combinations), and *gender* (two different combinations) (Table 2).^7^ We further varied the *number of job changes* of the hypothetical applicants (two different combinations).^8^ In this paper, we do not compare the effect of different unemployment timings,^9^ and we do not focus on the effect of the number of job changes. This results in a vignette universe of 252 vignettes, that is, our design has 252 possible combinations of signals. Based on pretest response rates, we decided to field a fraction of 180 vignettes allocated to 18 decks (10 vignettes per deck) and optimised this subset for maximal D-efficiency and minimal confounding with respect to the fractionalised universe of 180 vignettes and to the assignment of vignettes to decks (Auspurg and Hinz 2015). The allocation of nine vignettes to the vignette decks resulted from a randomisation. In every vignette deck, we embedded one ideal hypothetical CV with occupation-specific education and work experience, at least upper secondary education, no unemployment, and no frequent job changes. Our analysis shows that, when controlling for the ideal vignette, the results stay the same. Nationality (Swiss) and a total time of 5 years spent on the labour market (employed or unemployed) since leaving formal education—the mean age thereby depended on the level of education—were held constant in the experiment. An example of a fictive CV is shown in the Appendix: Fig. 1.

**Table 2:** Experimentally varied characteristics of the hypothetical applicants

Experimental variables	Levels of the experimental variables
Level and occupation-specificity of education and work experience	Lower secondary education and occupation-specific low-skill job experience (credentials and job titles according to the occupational field of the advertised job) Occupation-specific upper secondary education and occupation-specific middle-skill job experience (credentials and job titles according to the occupational field of the advertised job) Occupation-specific tertiary education and occupation-specific high-skill job experience (credentials and job titles according to the occupational field of the advertised job) Lower secondary education and non-occupational low-skill job experience (credentials and job titles from the retail sector) Non-occupation-specific upper secondary education and non-occupation-specific middle-skill job experience (credentials and job titles from the retail sector) Non-occupation-specific tertiary education and non-occupation-specific high-skill job experience (credentials and job titles from the retail sector) Lower secondary education and work experience in unqualified jobs (credentials according to the occupational field of the advertised job, and the job title “call-centre agent”) Occupation-specific upper secondary education and work experience in unqualified jobs (credentials according to the occupational field of the advertised job, and the job title “call-centre agent”) Occupation-specific tertiary education and work experience in unqualified jobs (credentials according to the occupational field of the advertised job, and the job title “call-centre agent”)
Duration and timing of unemployment	No unemployment 10 months of unemployment after graduation 20 months of unemployment after graduation 10 months of unemployment between jobs 20 months of unemployment between jobs 10 months of current unemployment 20 months of current unemployment
Gender	Male Female

The variable *specificity of education and work experience* reflects whether the applicants had trained and worked in one of the five defined occupations or in an unrelated occupational field. The variable has three categories: *occupation*-*specific education and work experience, non*-*occupation*-*specific education and work experience*, and *occupation*-*specific education and work experience in unqualified jobs*. To define suitable education credentials for vignettes with *occupation*-*specific education and work experience* in each of the five occupational fields, we used the official Swiss career-counselling webpage (Berufsberatung 2017), which provided detailed information about the required education and skills for specific occupations in Switzerland. The category *non*-*occupation*-*specific education and work experience* is operationalised by education credentials and respective work experience in the *retail* sector. The category *occupation*-*specific education and employment experience in unqualified jobs* represents job candidates who had educational credentials matching the occupational field of the advertised job position, but they were working as *call*-*centre agents.* In addition, we distinguished three different *levels of education*: lower secondary degree, upper secondary degree, and tertiary degree. In all cases, the skill level of *work experience* in the CV was defined so that it corresponded to the *level of education,* which means that a holder of a *lower secondary degree* was defined to have work experience in a *low*-*skill job*, a holder of an *upper secondary degree* in a *middle*-*skill job,* and a holder of a *tertiary degree* in a *high*-*skill job. Work experience* as a *call*-*centre agent* was an exception and could follow a *lower secondary, upper secondary*, or *tertiary education*. For example, a candidate with a BA degree (*tertiary degree*) in advanced mechanics (*educational specificity matching the occupational field)* would have job experience as a chief mechanic (*high*-*skill level job matching the occupational field*). In total, this gives us nine different combinations of education and work experience that differ with respect to occupation specificity and skill level.

To test the impact of different unemployment durations, we used 10 months to proxy *short unemployment* and 20 months to proxy *long unemployment*.^10^ A maximum of one period of unemployment could occur on the CV. The durations of unemployment were determined by considering the different unemployment rates in the four countries within the multinational project. While 10 and 20 months of unemployment could be regarded as rather long in Switzerland, such durations are reasonable in Bulgaria and, especially, in Greece. In the Swiss context, such a long period unemployment allows for a fairly conservative test of our null hypothesis. Or in other words, if no unemployment effect can be found despite the pronounced unemployment duration, shorter periods of unemployment are even less likely to cause any effects. The ex post created variable *profile match* measures whether a hypothetical CV contains the required educational background and work experience of the advertised job. *No match* applies if a CV does not contain the required occupation specificity (horizontal mismatch) or if the required level of education is not met (vertical mismatch). Finally, we control for gender (male = 1).

To further assess our hypotheses H2 and H3, three variables—*profile match*, *match in occupation specificity*, and *match in level*—were generated ex post by combining survey and experimental data.^11^ The survey data provided information about the education and job experience required by each advertised job in our sample, which we matched to our vignette data. For the variable *profile match*, a match is given if the vignette shows both the required occupation specificity and level in education and job experience. For *match in occupation specificity*, a match occurs if the vignette shows the required occupation specificity in education and job experience. And for *match in level*, a match is given if the vignette shows the required level in education and job experience.

In our design, employers are asked to rate the CV of ten hypothetical applicants—the vignettes—which are shown in a randomised order, on a scale from 0 to 10 with regard to the applicants’ chances of being considered for the specific position they are currently recruiting for. In addition to the vignette dimensions presented above, we controlled for the occupational field of the job position and the primacy effect with a dummy variable indicating the first and second vignette in the series of ten consecutively rated CV. Each vignette contained an intuitively accessible graphical representation of a short fictional CV (see Appendix: Fig. 1).

### Analytical strategy

To examine the effects of unemployment and our other experimental variables on the log-transformed rating^12^ of recruiters, we employed random-effects multilevel-linear regression models (see Auspurg and Hinz 2015). Every recruiter rated multiple vignettes, so we have to account for clustering at the level of the recruiters. Our analytical strategy accounts for the nested nature of our data through recruiter-level random effects and by employing cluster and heteroscedasticity-robust standard errors.

With the first two models, we examine H1, which states that *the chance of being considered for a job is negatively associated with the presence and duration of a period of unemployment*. In Model 1, we regress the vignette evaluations on unemployment while controlling for the occupational field of the job position, the applicants’ level of education and work experience, the occupation specificity of education and work experience, gender, and the vignette order. In Model 2, we differentiate between long and short unemployment.

In Model 3, 4, and 5, we add the *profile*-*match* variables to account for the match between the applicants’ profile (education and job experience) and the job requirements as well as the interaction of each match variable with unemployment. With Model 3, we are testing H2 (*the chance of being considered for a job is negatively associated with the presence of a period of unemployment specifically when the applicant’s profile meets the job requirements*). To test H3 (*the chance of being considered for a job is negatively associated with the presence of a period of unemployment specifically when the applicant shows occupation*-*specific educational background and work experience*), we compared the moderating effect of horizontal and vertical mismatch on unemployment scarring.

## Results

Due to the log-transformed dependent variable—the vignette rating—the coefficients are to be interpreted as approximate changes in the percentage points of the recruiters’ ratings with a unit change of the independent variable. Table 3 shows marginal effects for Model 1 and 2. The presence of unemployment in a CV reduces the rating by approximately 10% points (Model 1, Table 3). This corroborates hypothesis H1, which states that unemployment will negatively impact the chance of being considered for a job. We are controlling for *education and work experience*, the *occupational field* of the advertised positions, the *order effect*, and the *gender* of the applicants. The *educational background and job experience* of the applicants have significant impact on the recruiters’ ratings. In comparison to *lower secondary education and occupation*-*specific low*-*skill job experience* as the reference category, *occupation*-*specific upper secondary education and occupation*-*specific middle*-*skill job experience* as well as *occupation*-*specific tertiary education and occupation*-*specific high*-*skill job experience* significantly improve the ratings. *Non*-*occupation*-*specific education and work experience*, on the other hand, decreases the ratings regardless of the level of education and job experience. Applicants with *occupation*-*specific education and job experience as call*-*centre agents* are rated lower, but the negative effect is not significant for applicants who hold a *tertiary degree*. There are significant differences between the occupational fields of the advertised position (with *catering* as the reference category), which are not presented in the table. The significant *primacy effect* indicates that recruiters rated the first two CV presented to them less favourably than the others. We did not find a *gender* effect.

**Table 3:** Overall scarring effects of unemployment (standard errors are displayed in parentheses)

	Model 1	Model 2
	b (se)	b (se)
*Unemployment* (ref. no unemployment)	− 0.100*** (0.02)	/
*Duration of unemployment* (ref. no unemployment)		
Short unemployment	/	− 0.084*** (0.02)
Long unemployment	/	− 0.118*** (0.02)
*Type of education—work trajectory* (ref.: lower secondary education and occupation-specific low-skill job experience)		
Occupation-specific upper secondary education and occupation-specific middle-skill job experience	0.723*** (0.04)	0.726*** (0.04)
Occupation-specific tertiary education and occupation-specific high-skill job experience	0.573*** (0.05)	0.575*** (0.05)
Lower secondary education and non-occupation-specific low-skill job experience	− 0.519*** (0.04)	− 0.513*** (0.04)
Non-occupation-specific upper secondary education and non-occupation-specific middle-skill job experience	− 0.463*** (0.04)	− 0.457*** (0.04)
Non-occupation-specific tertiary secondary education and non-occupation-specific high-skill job experience	− 0.402*** (0.04)	− 0.400*** (0.04)
Lower secondary and call-centre job experience	− 0.432*** (0.04)	− 0.428*** (0.04)
Occupation-specific upper secondary education and call-centre job experience	− 0.115** (0.04)	− 0.112** (0.04)
Occupation-specific tertiary education and call-centre job experience	− 0.038 (0.05)	− 0.035 (0.05)
*Gender* (ref. female)	0.011 (0.01)	0.012 (0.01)
*Presentation order of the vignettes* (ref. 3rd to 10th vignette)	− 0.061*** (0.02)	− 0.060*** (0.02)
*Constant*	1.189*** (0.07)	1.186*** (0.07)
R^2^	0.340	0.341
Observations	6338	6338
Persons	638	638

The coefficients are log-transformed vignette ratings and can be interpreted as approximate changes in the percentage points of recruiters’ ratings with a unit change of the independent variable

^+^ p < 0.10, * p < 0.05, ** p < 0.01, *** p < 0.001

In Model 2, we allow for differences in ratings due to *short unemployment* and *long unemployment* (reference category: *no unemployment*). *Long and short periods of unemployment* are associated with respective decreases of 12 and 8% points in the ratings. The difference in the effect of *long and short periods of unemployment* is both substantive and significant (χ^2^(1) = 5.66, p < 0.05). These findings lend further support to H1. Periods of unemployment are globally associated with less favourable ratings. More precisely, long periods of unemployment will evoke a greater penalty than shorter periods of unemployment.

To test H2, we added the variable measuring of the (horizontal *and* vertical) *match of the applicants’ profiles with the job requirements* and the interaction term of *profile match* and *unemployment* in addition to the variables applied in Model 1 (Model 3, Table 4). When the applicants possess the required education and job experience (*match*), the rating increases by 59% points, whereas a period of unemployment reduces the rating by 7 percentage points. Periods of unemployment lead to an additional decrease of 6% points in the recruiters’ ratings if the candidate’s profile matches the job requirements, but this effect is no longer significant. *Occupation*-*specific tertiary education and occupation*-*specific high*-*skill job experience* decreases in its magnitude and lost its significance. *Gender* remains insignificant, and the order effect remains unchanged.

**Table 4:** Scarring effects of unemployment with profile match as moderator (standard errors are displayed in parentheses)

	Model 3	Model 4
	b (se)	b (se)
*Unemployment* (ref. no unemployment)	− 0.073*** (0.02)	− 0.010 (0.03)
*Match in applicant’s profile and job requirements* (ref. no match)		
Match in applicant’s profile and job requirements (occupation specificity and level)	0.593*** (0.06)	/
Match in applicant’s profile and job requirements (only in occupation specificity)	/	0.508*** (0.10)
Match in applicant’s profile and job requirements (only in level)	/	0.403*** (0.05)
*Unemployment and match* (ref. no unemployment and no match)		
Unemployment with match in applicant’s profile and job requirements (occupation specificity and level)	− 0.064^+^ (0.04)	/
Unemployment with match in applicant’s profile and job requirements (only in occupation specificity)	/	− 0.100** (0.04)
Unemployment with match in applicant’s profile and job requirements (only in level)	/	− 0.060^+^ (0.03)
*Type of education—work trajectory* (ref.: lower secondary education and occupation-specific low-skill job experience)		
Occupation-specific upper secondary education and occupation-specific middle-skill job experience	0.430*** (0.06)	0.514*** (0.05)
Occupation-specific tertiary education and occupation-specific high-skill job experience	0.058 (0.07)	0.215*** (0.06)
Lower secondary education and non-occupation-specific low-skill job experience	− 0.526*** (0.04)	− 0.113 (0.10)
Non-occupation-specific upper secondary education and non-occupation-specific middle-skill job experience	− 0.455*** (0.04)	− 0.247* (0.10)
Non-occupation-specific tertiary secondary education and non-occupation-specific high-skill job experience	− 0.406*** (0.04)	− 0.326** (0.11)
Lower secondary education and call-centre job experience	− 0.428*** (0.04)	− 0.04 (0.10)
Occupation-specific upper secondary education and call-centre job experience	− 0.127** (0.04)	0.083 (0.10)
Occupation-specific tertiary education and call-centre job experience	− 0.073 (0.05)	0.006 (0.11)
*Gender* (ref. female)	0.007 (0.01)	0.006 (0.01)
*Presentation order of the vignettes* (ref. 3rd to 10th vignette)	− 0.056** (0.02)	− 0.056** (0.02)
Constant	1.176*** (0.07)	0.716*** (0.12)
R^2^	0.356	0.351
Observations	6298	6298
Persons	634	634

The coefficients are log-transformed vignette ratings and can be interpreted as approximate changes in the percentage points of recruiters’ ratings with a unit change of the independent variable

^+^ p < 0.10, * p< 0.05, ** p< 0.01, *** p< 0.001

To differentiate between the moderating effect of a *(mis*-*)match in the occupation specificity* of education and job experience from the moderating effect of a *(mis*-*)match in the level* of education and job experience, we separate the horizontal and vertical(mis-)match effects in Model 4. Both a *match in occupation specificity* and a *match in level* significantly increase the rating by 51 and 40% points, respectively. The *unemployment* effect is neither significant nor substantive when there is *no match* between the applicants’ profiles and the job requirements. Also, the interaction term of *level match* and *unemployment* does not become significant, indicating that the size of the unemployment effect does not depend on the required level in education and job experience. By contrast, the significant interaction term of *occupation*-*specificity match* and *unemployment* suggests a substantive scarring effect of unemployment of approximately—10% points, which is limited to applicants with the required occupation specificity in education and job experience.

Looking at the control variables after differentiating how well applicants’ profiles match the job requirements, the previous effects of some control variables, such as *call*-*centre job experience*, notably decreased. The *gender* and *order effect* are again largely unchanged.

## Discussion

We have attempted to contribute to the debate on mismatch and unemployment by analysing how a (mis-)match between an applicant’s profile and the job requirements moderates unemployment scarring and by differentiating between mismatch in occupation specificity and mismatch in educational level. The presented results largely support our hypotheses, which allows us to make two contributions. First, we have found that the recruiter rating of job applicants’ CV decreases with increasing lengths of unemployment. This finding supports the first hypothesis, which states that longer periods of unemployment have stronger negative signalling effects than shorter periods. A possible explanation could be that employers suspect human-capital depreciation to advance with increasing durations of labour-market inactivity. Another explanation could be that employers interpret long-term unemployment as a strong negative signal indicating problematic traits of the applicant, some of which may not be observable during recruitment. This is especially likely in the Swiss context where the youth unemployment rate is low and long-term unemployment is rare. Our results are in line with previous similarly designed studies that also found evidence for negative duration dependence (Eriksson and Rooth 2014; Kroft et al. 2013; Oberholzer-Gee 2008). But while we have found that 20-month periods of unemployment are rated significantly worse than 10-month periods, Kroft et al. (2013) suggested that the negative effects of unemployment on callback rates only increase during the early phase of unemployment and stabilise after the first 8 months. And Oberholzer-Gee (2008) demonstrated that unemployment had no effect on the callback rate for fictive applicants with 18 months of unemployment, and that the unemployment effect only kicks in when unemployment lasts longer than two-and-a-half years. Eriksson and Rooth (2014), on the other hand, found the negative unemployment effect to occur after 9 months but only in low- and middle-skill jobs and not in high-skill jobs. In contrast, our findings differ from studies that have not found evidence for negative duration dependence (Nunley et al. 2017, Farber et al. 2015). Possible reasons for the minor and major discrepancies are most likely due to the different experimental settings, sample characteristics, and labour-market conditions. Regarding the operationalisation of unemployment, different durations were employed in the literature. While Eriksson and Rooth (2014) used 9 months to proxy long periods of unemployment, Oberholzer-Gee (2008) used up to 30 months, and we tested with 10 and 20 months. Furthermore, while Kroft et al. (2013) sampled for low- and middle-skill jobs in major US cities and excluded jobs requiring advanced degrees, Oberholzer-Gee (2008) restricted his sample to administrative-assistant positions in Switzerland. Nunley et al. (2017) sampled graduate jobs requiring college degrees in seven US cities. Similarly, Farber et al. (2015) limited their sample to white-collar office jobs in eight US cities. In our study, we collected vacancies in five occupational fields covering low-, middle-, and high-skill jobs in German-speaking Switzerland. Lastly, the fictive CV in these studies differ with regard to gender, age, education, and ethnicity.

As a second contribution, we have found that a detailed operationalisation of educational background and work experience helps to explain the heterogeneity in recruiters’ assessments of young job applicants. Applicants with occupation-specific education and work experience are evaluated significantly better than applicants who do not have occupation-specific education and have worked in an unrelated occupation. Also, compared to candidates with upper secondary or tertiary degrees, unqualified applicants with a compulsory education have much lower chances of being considered for the advertised position.^13^ Interestingly, job experience as a call-centre agent decreases the recruiters’ ratings of applicants with lower secondary or upper secondary degrees, but for the tertiary degree holders, work experience in an unqualified job does not lead to a decrease in the recruiters’ evaluations. This may indicate that employers believe that tertiary degree holders have already demonstrated their potential abilities and productivity by graduating from higher education. Working as a call-centre agent could therefore be considered a (temporary) voluntary choice.

We further analysed the interaction effect of unemployment and match between the applicants’ profiles and the job requirements. We argued that in the Swiss context, where the trust of recruiters in credentials is strong, the negative effect of unemployment only unfolds in situations where the match between the applicant’s profile and the job requirements is given and that unemployment does not matter if applicants do not have a matching profile. In line with hypothesis (H2), we found stronger unemployment scarring for applicants with matching profiles than for applicants who do not meet the basic job requirements. But this difference is not statistically significant (significance at a level of p < 0.10).

This non-significant result required further investigation. We proposed differentiating between horizontal and vertical (mis-)match between the applicants’ profiles and the job requirements in order to disentangle the (mis-)match in level from the (mis-)match in occupation specificity. It then became apparent that in the Swiss context, unemployment only scars if the applicant has occupation-specific education and job experience. In all other cases, unemployment does not decrease the perceived chances of being considered for the advertised position. This result corroborates our third hypothesis (H3). The previously insignificant effect of unemployment for applicants with an adequate educational background and job experience might partly be explained by insufficient differentiation between horizontal and vertical profile (mis-)match. Within the strongly occupationally segmented Swiss labour market, occupation-specific training and work experience seems to be crucial for job applicants. If applicants do not meet these conditions, unemployment does not further decrease their chances of being considered for the job. If, however, applicants have passed the first threshold, unemployment does become a factor that has a negative impact on recruiters’ judgment. These findings seem plausible for a country in which on-the-job training is not the predominant form of skill acquisition. Employers rather expect applicants to possess the knowledge and skills relevant for the specific occupation. If applicants were trained in an unrelated occupation, unemployment is not associated with human-capital depreciation since they are not endowed with the required specific human capital in the first place. To validate whether our findings are specific to the context of a strongly VET-focused education system and occupationally segmented labour market, further studies in other countries with comparable economic conditions but different education systems and labour-market structures would be necessary.

## Conclusion

We found that the presence of unemployment decreases the perceived suitability of applicants for vacancies and that the scarring effect of unemployment increases with its duration. Moreover, our paper contributes to the literature by demonstrating that whether unemployment scarring occurs depends on how well the applicant’s profile meets the job requirements. In a country with a strongly standardised VET system, the scarring effect of unemployment only unfolds if the applicant has received the required occupation-specific training and has worked in the appropriate occupational field. If candidates do not have the required occupation-specific education and job experience, they have much lower chances of being considered for the position, and unemployment does not additionally scar their chances. Overall, our findings suggest that recruiters’ expectations of human-capital depreciation during unemployment is a driving mechanism in causing unemployment scarring in the Swiss context: obviously, recruiters can only associate unemployment with skill depreciation if applicants have actually acquired the relevant skills in the first place. In this sense, our results indicate that recruiters suspect an undesirable loss of *occupation*-*specific* human capital if applicants have been unemployed for some time. By contrast, we have no evidence that unemployment serves as a *general signal* of undesirable applicant characteristics since applicants without the requested occupation-specific skills are hardly affected by unemployment scarring at all. We do find support for signalling theory, however, when it comes to how employers use education degrees and previous work experience as hiring criteria: the strong positive effect of matching education degree and job title can be interpreted as due to the employers’ strong trust in credentials. This is plausible in an education system that is predominated by the VET system.

A limitation of our study is that we asked the recruiters to evaluate fictive applicants’ chances of being considered for advertised jobs, and their evaluations may therefore deviate from their actual hiring behaviour. Furthermore, our findings are confined to the German-speaking region of the Swiss labour market, which shows a relatively low youth unemployment rate compared to other European countries that have been severely hit by the recent Great Recession. For labour-market contexts with increasing employability requirements, unemployment seems to possess a signalling power in the eyes of employers. Further research is needed to understand how the occupational match of job applicants and jobs impacts unemployment scarring in countries where general education predominates instead of VET and the linkage of education and employment is weaker.

## Appendix

See Table 5, Fig. 1.

**Table 5:** Response rates in the occupational fields

Occupational field	Absolute response rate (N)	Response rate (%)
Mechanics	129	30.6
Finance	126	31.6
Nursing	205	45.9
Catering	115	29.6
ICT	164	35.6

## Footnotes

1 About two thirds of lower secondary-school graduates entered VET education in 2013, and about one third enrolled in general education. These proportions have continued to remain constant over recent years (Schellenbauer et al. 2010).

2 NEGOTIATE is a Horizon 2020 project involving ten participating countries (https://negotiate-research.eu/).

3 Swiss Job Market Monitor (2015) annually surveys comprehensive random samples of job ads published in Switzerland and has an internationally unique database on vacancies and job profiles dating back to 1950.

4 Results from yearly firm surveys indicate that in 2014, a markedly rising share of over 50% of all vacant jobs were advertised on job portals (Swiss Job Market Monitor 2015: 28).

5 Of the 2118 recruiters, 580 (27%) completed the survey.

6 The experimental variables are explained in more detail in Shi et al. (2015).

7 *Gender* is not an analytical focus of this contribution and will only be used as a control variable, but it was implemented as an experimental variable in the overall study.

8 Different frequencies of job changes in the applicants’ CV make it possible to measure the effect of job hopping on recruiters’ evaluations.

9 Preliminary analyses indicate that the reported unemployment effects in this study do not depend on the different timings of unemployment.

10 There is no agreement in the literature on the point of time when the scarring effect of unemployment becomes significant. Depending on country-specific settings, short unemployment is defined as between 6 and 10 months (Eriksson and Rooth 2014; Kroft et al. 2013; Oberholzer-Gee 2008).

11 A match is given (*match *= 1) if the level and field of education and work experience on the fictional CV meet the corresponding job requirements as captured by the recruiter survey.

12 Given the skewed distribution of the ratings, we took their natural log to approach approximately normally distributed residuals.

13 It should be noted that we only sampled for jobs requiring formal credentials or certificates. The penalisation of those with less education is therefore not a surprise.
